# 

**Published:** 1906-09

**Authors:** 


*The following donations have been received since the publication of the List in June:*

*August 31st, 1906.*

Associated Physicians of Long Island (1) ... ... 1 volume.

L. M. Griffiths (2) ... ... ... ... ... ... ... 7 volumes.

Manchester Medical Society (3) ... ... ... ... 6 volumes.

Middlesex Hospital (4) ... ... ... ... ... ... 1 volume.

Rhode Island Medical Society (5) ... ... ... ... 1 volume.

Unbound periodicals have been presented by the Manchester Medical Society.

### SIXTY-FIRST LIST OF BOOKS.

The titles of books mentioned in previous lists are not repeated.

The figures in brackets refer to the figures after the names of the donors, and show by whom the volumes were presented. The books to which no such figures are attached have either been bought from the Library Fund or received through the *Journal*.

**Allbutt, W. S. Playfair and T. W. Eden, T. C.** [Eds.] *A System of Gynæcology* ... ... ... ... ... ... [2nd Ed.] 1906

**Ball, J. B.** ... ... *Diseases of the Nose and Pharynx* ... ... ... 5th Ed. 1906

**Berkeley, C.** ... *Handbook for Midwives and Maternity Nurses* ... ... 1906

**Blackham, R. J.**... *The Care of Children* ... ... ... ... Revised Ed. [1906]

**Brend, W. A.** ... *Handbook of Medical Jurisprudence* ... ... ... ... 1906

**Buchanan, A. M.** *Manual of Anatomy*, Vol. I. ... ... ... ... ... 1906

**Calder, A. B.** ... *Lectures on Midwifery for Midwives* ... ... ... ... 1906

**Dalgado, D. G.** ... *The Climate of Lisbon* ... ... ... ... ... ... ... 1906

**Eden, T. C. Allbutt, W. S. Playfair and T. W.** [Eds.] *A System of Gynæcology* ... ... ... ... ... ... [2nd Ed.] 1906

**Elliott, M. and G.** *Public Health Legislation* ... ... ... ... ... ... 1906

*Encyclopædia and Dictionary of Medicine and Surgery* (Ed. by J. W. Ballantyne). Vol. I.... ... ... ... ... ... [N.D.]

**Hewlett, C. G. Moor and R. T.** *Applied Bacteriology* ... ... ... 3rd Ed. 1906
**Lovibond, J. W....** *Colour Phenomena* ... ... ... ... ... ... ... ... 1905
**Luke, T. D.** ... *Guide to Anæsthetics* ... ... ... ... .. 3rd Ed. 1906
**McCrudden, F. H.** *Uric Acid* ... ... ... ... ... ... ... ... ... ... ... [1905]
**Marsden, R. W.** *Hints on the Management of the Commoner Infections* ... 1906
**Moor and R. T. Hewlett, C. G.** *Applied Bacteriology* ... ... ... 3rd Ed. 1906
**Nunneley, F. P.** *Aneurysm of the Abdominal Aorta*... ... ... ... ... 1906
**Playfair and T. W. Eden, T. C. Allbutt, W. S.** [Eds.] *A System of Gynæcology* ... ... ... ... ... ... [2nd Ed.] 1906
**Rawling, L. B....** *Muscles and Nerves* ... ... ... ... ... ... ... ... ... 1906
**Schreber, M.** .. *Medical Gymnastics* (Trans. by H. Skelton) ... (2) 1856
*Selected Essays on Syphilis and Small-Pox* (Ed. by A. E. Russell) ... ... 1906
**Symonds, J. A. ...** *The Principles of Beauty* ... ... ... ... ... ... ... (2) 1857
**Wheeler, W. I. de C.** *Operative Surgery*... ... ... ... ... ... ... ... 1906

### TRANSACTIONS, REPORTS, JOURNALS, &c.

American Pediatric Society, Transactions of the ... ... Vol. XVII. 1906
Antiseptic, The .. ... ... ... ... ... ... ... ... ... ... ... Vol. II. 1905
Archives of the Middlesex Hospital ... ... ... ... ... (4) Vol. VII. 1906
Archivio di Ortopedia ... ... ... ... ... ... ... ... ... ... ... ... 1905
Associated Physicians of Long Island, Transactions of the... (1) Vol. VII. 1906
Australasian Medical Gazette, The ... ... ... ... ... Vol. XXIV. 1905
Boston Medical and Surgical Journal, The (3) Vols. XXIII., 1841, LXX.–LXXIII., 1864-66
British Medical Journal, The ... .. ... ... ... ... ... ... ...Vol. I. for 1906
Bulletin de l'Académie royale de Médecine de Belgique ... Tome XIX. 1905
California State Journal of Medicine, The ... ... ... ... Vol. III. 1905
College of Physicians of Philadelphia, Transactions of the Vol. XXVII. 1905
Columbia University, Studies from the Department of Pathology of the College of Physicians ... ... ... ... ... ...Vol. X. 1904-05
Dublin Obstetrical Society, Proceedings of the ... ... ...(2) 1872, 1875, 1876
Écho médical du Nord, L' ... ... ... ... ... ... ... ... Tom. IX. 1905
Edinburgh Medical Journal, The ... .. ... ... ... N.S., Vol. XIX. 1906
Glasgow Medical Journal, General Index to, 1828-88 ... ... ... (3) 1889
Johns Hopkins Hospital Bulletin, The ... ... ... ... ... Vol. XVI. 1905
Journal of the American Medical Association, The ... ... Vol. XLIV. 1905
Lancet, The ... ... ... ... ... ... ... ... ... ... ... ...Vol. I. for 1906
Liverpool Medico-Chirurgical Journal, The ... ... ... Vol. XXIV. 1904
Local Government Board. Report on Sewage Disposal ... ... ... (2) 1876
"Medical Annual" Synoptical Index, 1899-1904 ... ... ... ... ... [N.D.]
Medical Chronicle, The ... ... ... ... ... ... 4th ser., Vol. X. 1905-06
Progressive Medicine ... ... ... ... ... ... ... ... ... Vol. II. 1906
Rhode Island Medical Society, Transactions of the (5) Vol. VII. Pt. 2 1906
Royal Academy of Medicine in Ireland, Transactions of the Vol. XXIV. 1906
Scottish Medical and Surgical Journal, The ... ... ... Vol. XVIII. 1906
South African Medical Record ... ... ... ... ... ... ... ... Vol. III. 1905
Treatment ... ... ... ... ... ... ... ... ... ... ... Vol. IX. 1905-06
University of Penna. Medical Bulletin ... ... ... ...Vol. XVIII. 1905-06

### Local Medical Notes.

**University College, Bristol.**—Students of the College have recently passed the following examinations:—

UNIVERSITY OF LONDON.—*M.B., B.S.*—J. F. Blackett, J. B. V. Watts, J. S. Avery (*Group II.*) *Intermediate Examination:* T. B. Dixon, E. R. Holborow, C. A. Joll, B.Sc., F. S. Scott.

CONJOINT BOARD OF ENGLAND.—*First Examination: Chemistry and Physics*—T. E. Ashley, H. H. Hiley, S. Marle, W. E. Neale, V. Pinnock. *Practical Pharmacy:* C. H. Hart, T. S. Rippon. *Elementary Biology*—W. E. Neale, V. Pinnock. *Second Examination: Anatomy and Physiology*—E. C. Davies, P. C. Field, F. C. Morgan, F. C. Nicholls, A. J. O. Wigmore. *Final Fxamination: Medicine*—W. W. King*, R. E. Thomas*, V. B. Green-Armytage*, H. K. Salisbury*. *Surgery*—C. S. Rivington*, V. B. Green-Armytage*. *Midwifery*—C. E. K. Herapath.

L.D.S., ENG.—*Chemistry and Physics*—W. E. Drummond, F. J. Hayman, S. V. Shrimpton.

L.S.A.—*Surgery, Medicine: Section II.*—P. Moxey*, E. J. F. Thomas*. *Forensic Medicine*—E. V. Connellan.

TRINITY EXAMINATION (1906) FOR CALL TO BAR.—*Constitutional and Roman Law*—C. Corfield, M.R.C.S.

**Bristol Royal Infirmary.**—*Junior House Surgeon:* A. J. Wright, M.R.C.S., L.R.C.P. *Casualty Officer:* V. B. Green-Armytage, M.R.C.S., L.R.C.P.

**Bristol General Hospital.**—*House Physician:* L. N. Morris, M.R.C.S. *House Surgeon:* F. G. Bergin, M.R.C.S. *Casualty Officer:* W. J. H. Pinniger, M.B., B.S., Lond. *Assistant House Physician:* M. R. O. Wilson, M.R.C.S.

**Weston-super-Mare Hospital.**—Francis Shingleton Smith, M.C. (Cantab.), M.R.C.S., L.R.C.P., has been appointed House Surgeon.

**Winsley Sanatorium.**—L. B. Eskell, L.D.S., R.C.S.I., has been appointed Honorary Dental Surgeon.

**Long Fox Lecture.**—The third "Long Fox Lecture" will be given by Dr. Alfred J. Harrison, Consulting Physician to the Bristol General Hospital. The title and date of the Lecture will be announced in due course.

**To Old Bristol Students.**—On August 19th, at Old Park, there passed away, at the ripe age of 88, an individual who had in times now a little remote a great deal to do with the junior members of the profession in Bristol. William Fitz-

* Completes Examination.

Patrick, known personally to some and by reputation to all students of the Medical School by the shorter name of Fitz, was a man of striking personality. He was by appointment porter, but he was in reality a great deal more. He it was who explained and amplified the prospectus and the Dean's welcome to any embryo specialist and his parent. He kept the museum, he injected the bodies, he macerated the remains from the dissecting room, and had generally for sale complete sets of carpal and tarsal bones and often larger ones. He especially prized a sphenoid or an ethmoid, and could with assurance and more or less accuracy demonstrate the articulations of each. He mounted the wet specimens, filtered the spirit, and varnished the edges of the cover glasses. He had ready for every lecture the diagrams that he knew would be wanted; and, in fact, retained the power that his early maritime training gave him of being a handy man. A quarter of an hour after a lecture had begun Fitz would walk in with a long, untidy book in his hand and mark down the names of those present; and should any of us students suspect that we had not put in the number of attendances that would entitle us to be signed up for any course, a little pecuniary arrangement with Fitz generally brought about the required result. He used to call us all by our surnames without any prefix; and I, the writer of these reminiscences, possess a letter from him which he sent me one night after a faculty meeting when the prize marks were added up. It runs: "Dear P.,—I here you have again took the 2nd years prize. Yours Fitz." He held sway in the School till its removal about 1880 to its present site, and then it was thought that some sacrifice to more modern methods should be made, accordingly Fitz had to retire, and he has lived since in a house close to the Old Park Medical School gates. He was a most interesting and original person, of kindly disposition and with a retentive memory, so that he could tell many stories about the students and lecturers of the quarter of a century during which he was at the School. He will never be forgotten by those with whom he had to do.

